# Glutathione and Nitric Oxide: Key Team Players in Use and Disuse of Skeletal Muscle

**DOI:** 10.3390/nu11102318

**Published:** 2019-09-30

**Authors:** Sara Baldelli, Fabio Ciccarone, Dolores Limongi, Paola Checconi, Anna Teresa Palamara, Maria Rosa Ciriolo

**Affiliations:** 1 IRCCS San Raffaele Pisana, Department of Human Sciences and Promotion of the Quality of Life, San Raffaele Roma Open University, 00166 Rome, Italyfabio.ciccarone83@gmail.com (F.C.);; 2 IRCCS San Raffaele ‘La Pisana’, 00166 Rome, Italy; annateresa.palamara@uniroma1.it; 3 Department of Public Health and Infectious Diseases, Sapienza University of Rome, 00185 Rome, Italy; 4 Department of Biology, University of Rome “Tor Vergata”, 00133 Rome, Italy

**Keywords:** atrophy, exercise, oxidative stress, nitrosative stress, inflammation

## Abstract

Glutathione (GSH) is the main non-enzymatic antioxidant playing an important role in detoxification, signal transduction by modulation of protein thiols redox status and direct scavenging of radicals. The latter function is not only performed against reactive oxygen species (ROS) but GSH also has a fundamental role in buffering nitric oxide (NO), a physiologically-produced molecule having-multifaceted functions. The efficient rate of GSH synthesis and high levels of GSH-dependent enzymes are characteristic features of healthy skeletal muscle where, besides the canonical functions, it is also involved in muscle contraction regulation. Moreover, NO production in skeletal muscle is a direct consequence of contractile activity and influences several metabolic myocyte pathways under both physiological and pathological conditions. In this review, we will consider the homeostasis and intersection of GSH with NO and then we will restrict the discussion on their role in processes related to skeletal muscle function and degeneration.

## 1. Introduction

γ-l-glutamyl-l-cysteinyl-glycine (glutathione, GSH) is the most versatile non-enzymatic antioxidant involved in cell signaling, detoxification and oxy-radical scavenging activity. Its structure, cellular concentration and the systems deputed to its synthesis, degradation and regeneration concur to these different functions. For instance: (i) GSH synthesis is a two-step process mediated by cytosolic enzymes (a faster process with respect to a gene-mediated one); (ii) the peculiar iso-peptide bond of the glutamate makes GSH a protease-resistant peptide; (iii) the NADPH-reductase systems guarantee the maintaining of a high ratio between reduced (GSH) and oxidized (GSSG, GSSR) forms under physiological conditions. Alteration of such ratio during oxidative burst can modulate redox signaling pathways related to various cellular processes including proliferation, growth and apoptosis [1].

**Figure 1:**
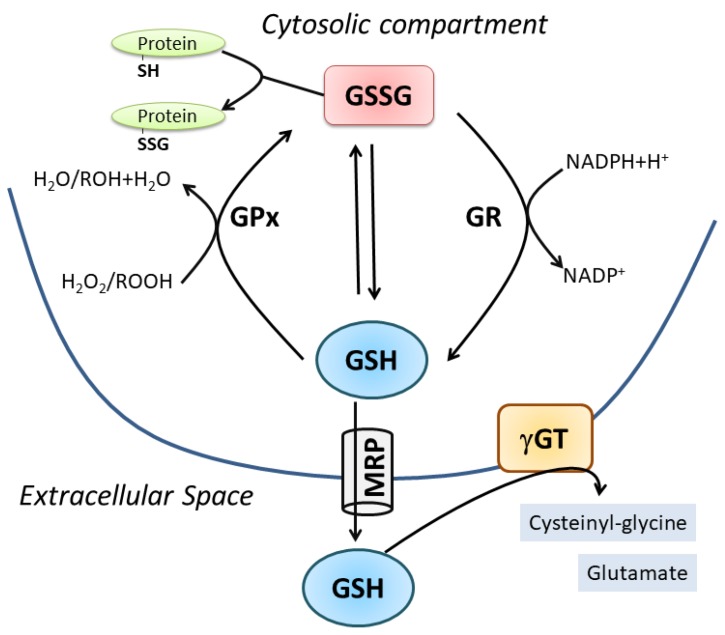
Cellular GSH homeostasis. For details see the text.

Two ATP-dependent cytosolic enzymes are responsible for the de novo synthesis of GSH: glutamate-cysteine ligase (GCL) also named γ-glutamylcysteine synthase (GCS) and glutathione synthase (GS). The first is the rate-limiting enzyme formed by a modulatory or light subunit (GCLM) and a catalytic or heavy subunit (GCLC). Using the ATP hydrolysis, GCLC is able to shape an iso-peptide bond between the γ-carboxyl of glutamate and the amino group of cysteine. GS catalyzes the addition of glycine to γ-glutamylcysteine created by GCLC to form GSH [1]. GCLC is transcriptionally regulated by Nuclear factor (erythroid-derived 2)-like 2 (NFE2L2), a transcription factor that regulates a wide array of antioxidant responsive element-driven genes in various cell types [2]. GSH degradation occurs in the extracellular space by the enzyme γ-glutamyl-transpeptidase (γ-GT), which cleaves GSH into cysteinyl-glycine (Cys-Gly) and glutamate. Thus, systemic GSH homeostasis relies on its intracellular synthesis, export and degradation outside the cell in a cycle with a half-life of 2–3 h [3]. Intracellularly, GSH is present in a dynamic equilibrium with its oxidized forms, mainly GSSG, which is the product of a disulfide bridge between two molecules of GSH directly produced upon oxidative burst or by the activity of the antioxidant enzyme glutathione peroxidase (GPx). Subsequently, GSSG is converter to GSH through the glutathione reductase (GR). GSSR represents a less concentrated form deriving from mixed disulfide formation with protein thiols (protein S-glutathionylation); however, this oxidized form is pivotal in redox signaling pathways representing a reversible posttranslational modification of proteins [4]. The value of GSH/GSSG ratio is universally accepted as index of cell redox status because of the scavenging effect of GSH against several reactive oxygen species (e.g., hydroxyl radical: OH, superoxide anion: O_2_^−^, hydrogen peroxide: H_2_O_2_, lipid peroxyl radical: HOO), either directly or through the activity of antioxidant enzymes, mainly the glutathione peroxidases. GSH homeostasis is fundamental for cell metabolism and function as stated by the association of altered GSH levels with numerous conditions and pathologies such as aging, neurodegeneration, cancer, inflammation and muscle degeneration (Figure 1) [5,6,7,8].

GSH plays an important role also in the field of nitric oxide (NO) physiology and pathology [9,10,11]. NO is a gaseous free radical synthesized by NO synthases (NOSs) starting from L-arginine, NADPH and oxygen. NO is a highly reactive molecule that despite being potentially toxic is implicated in a wide range of physiological processes, such as neurotransmission, vascular tone regulation, musculature contraction/relaxation and platelet aggregation [12,13,14]. Furthermore, it is an integral part of the inflammatory response to pathogens and cancer cells [15,16]. This versatility of NO mainly derives from its complex chemistry. Depending on its distribution and reactants, NO can exist in different redox forms with distinct and highly reactive properties, such as nitroxyl (HNO), oxides (NO_2_, N_2_O_4_ and N_2_O_3_), peroxynitrite (ONOO^·−^) and S-nitrosothiols (RSNO) [17].

**Figure 2:**
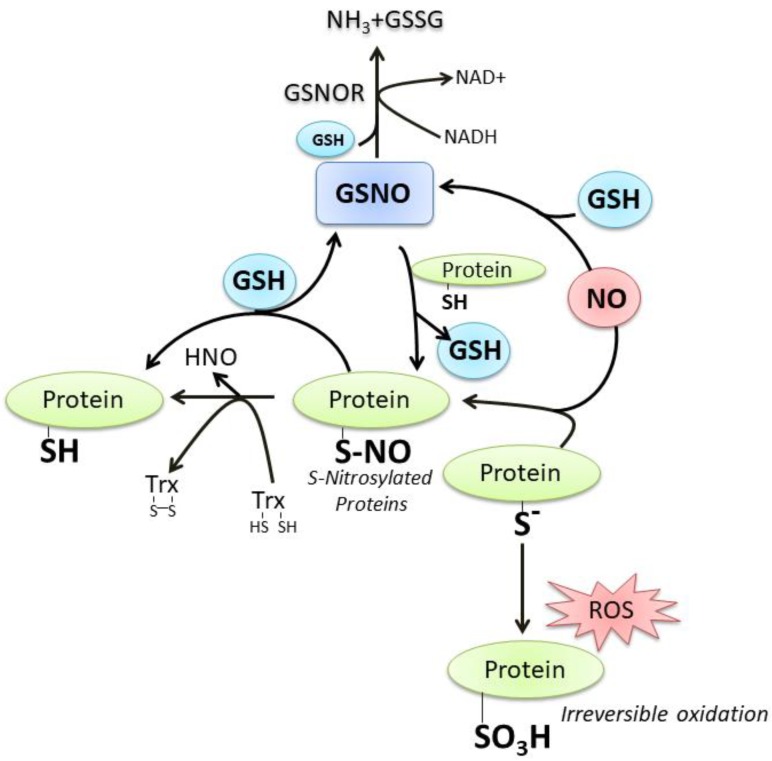
The crosstalk between GSH and NO. For details see the text.

GSH and NO can easily interact at different levels; a number of still debated mechanisms commit NO to react with GSH with the formation of S-nitrosoglutathione (GSNO), which is considered an endogenous NO reserve that contributes to the intracellular regulation of nitrosative stress [18]. In fact, we and other researchers demonstrated that neuronal NOS (nNOS) overexpression or treatments with NO donors result in intracellular GSH increase, a process fundamental to counteract neuronal death [11,19,20]. Thiol of “reactive cysteine” on proteins (P-SH) is another important target of reactive oxygen/nitrogen species (ROS/RNS) signaling pathway because it can be oxidized at a different extent up to the sulfonic form (P-SO_3_H), an irreversible oxidation product. On the contrary, the sulfenic form (P-SOH) represents the most frequent and reversible modification under physiological ROS/RNS flux. It can react with NO to produce the S-nitrosylation derivative (P-SNO), which seems to be implicated in the majority of long-range NO-cellular redox processes. The P-SNO can be reconverted to P-SH by thiol-dependent systems, among which GSH plays a prominent role through the translocation reaction of NO that generates GSNO [18,21] (Figure 2). Based on this evidence, it is conceivable to link the majority of NO effects on the availability and redox status of GSH.

Skeletal muscle is one of the tissues that actively produce high levels of ROS and NO under physiological condition, especially under physical exercise, and these reactive species are suggested to be de-regulated during muscle aging and pathological processes [8,22,23]. Several sources of ROS exist in the muscle (mitochondria, NADPH oxidases, phospholipase A2, xanthine oxidase) and NO is continuously produced at low extent also in muscle resting, while its production is increased during contractile activity. Moreover, skeletal muscle represents the largest pool of GSH (5 mM) in the human body since muscle accounts for about 40% of body mass [24]. GSH plays an essential role in protein catabolism and oxidative/nitrosative stress that is induced by intense physical exercise or injury. In fact, skeletal muscle has been reported to increase the uptake of GSH from the plasma to counteract the excessive production of ROS occurring after prolonged and intense physical exercise [25]. The connection between GSH and NO will be examined in muscle as regulators of redox-mediated signaling and other processes related to oxidative/nitrosative stress throughout this review.

## 2. Oxidative/Nitrosative-Mediated Signaling and Stress as the Conditions Where NO Mainly Intersects GSH

Oxidative/nitrosative stress is properly “an imbalance between oxidants and antioxidants in favor of the oxidant species, potentially leading to damage” [26,27]. ROS are by-products of the sequential one-electron reduction of molecular oxygen, which includes radical and non-radical species. RNS are a group of reactive compounds that include NO, which is physiologically produced by NOSs, a NADPH-dependent family of enzymes present in most cells and tissues [28]. To date, three main isoenzymes belonging to this family have been cloned and purified: nNOS, endothelial NOS (eNOS) and inducible NOS (iNOS) [29]. The first two are constitutively expressed and calcium (Ca^2+^)-dependent enzymes. nNOS is mainly expressed in cells of neuronal origin where NO functions as neuromodulator and neurotransmitter in the central and peripheral nervous system. Notably, the nNOS isoform is also present at the skeletal and cardiac muscle level, namely the nNOSμ [30,31]. It is characterized by a 34-amino acid insert that arises from the alternative splicing of nNOS pre-mRNA between exons 16 and 17. The catalytic activity is similar to that of nNOS and its expression coincides with the formation of differentiated myotube in cultured cells. eNOS is localized in the endothelium, in cardiomyocytes and adipocytes. It principally participates in the regulation of blood pressure and vascular tone. Instead, iNOS is a Ca^2+^-independent and cytokine-inducible enzyme, expressed in macrophages during inflammation and tissue injury [32]. In recent years, the importance of the fourth isoform of NOS has also emerged: the mitochondrial NOS (mtNOS) [33]. It is localized in mitochondria of several tissues, including skeletal muscle, where it regulates O_2_ consumption [34].

NO is a hydrophobic gas molecule with a high diffusion coefficient but a short life-time mainly due to its rapid reaction with oxygen and multiple cellular components. Therefore, as in the case of ROS, low and regulated production of NO is associated with paracrine signaling processes whereas its overproduction can cause damage to biological macromolecules and determine many pathological states [18,23,35]. In this regard, the product of NO reaction with O_2_^−^ leads to the formation of ONOO^·−^, which is a strong oxidant that reacts with lipids, DNA, and proteins via direct oxidative reactions or via indirect, radical-mediated mechanisms [36].

NO avidly reacts with transition metals; its rate constant of binding to iron(II) heme groups is >10^8^ M^−1^s^−1^. Indeed, ferrous heme can bind to numerous gaseous ligands including oxygen, carbon monoxide and NO, a paramount process through which heme entraps gas for several cellular functions. Among the heme-proteins, soluble guanylyl cyclase (sGC) is the principal and most studied receptor for NO: This enzyme plays a central role in blood pressure regulation and several other physiological actions. The binding of NO to a ferrous heme in its β subunit increases both catalytic rate and active site affinity for the substrate [37].

Along with the formation of GSNO after reaction with GSH, NO can readily react with the sulfhydryl group (-SH) of protein cysteine residue to form S-nitrosocysteine that consists of a redox-mediated posttranslational modification. S-nitrosylation is a reversible process that can affect the structure/function of the target cysteine, therefore it can be considered a way through which redox-mediated cellular signal is transduced as well as an endogenous reservoir for NO endocrine functions. De-nitrosylation, the process reforming the thiol group, requires a thiol-based molecular mechanism centered on GSH and thioredoxin (Trx) by means of non-enzymatic thiol exchange. Trx is a small molecular weight protein with reactive cysteine residue performing reduction of oxidized thiol-derivatives; in the de-nitrosylation process two vicinal cysteines in the protein become oxidized while the nitrosothiol is reduced producing free nitroxyl (HNO) or NO, then Trx is efficiently reduced by the NADPH-dependent activity of Trx reductase enzyme. GSH-dependent protein de-nitrosylation occurs through a nitrosylation exchange mechanism that generates GSNO, which can be a substrate of the enzyme S-nitrosoglutathione reductase (GSNOR) that finally produces GSSG and ammonia (NH_3_) [38,39,40]. A great number of proteins (about 240) are found to undergo S-nitrosylation: 70% of them were identified under physiological conditions, 12% under various pathological conditions and 15% may be ascribed to both conditions. Most of these proteins are located in the mitochondrial intermembrane space and the inner mitochondrial membrane. As regards the biological function, a significant proportion of them is involved in the generation of precursor metabolites and energy, including glucose metabolism, and oxidative phosphorylation [38].

Another important aspect related to the crosstalk between GSH and NO refers to the antioxidant function. In fact, while GSH is a canonical antioxidant a role for NO in this context is not straight and mainly related to its “chemistry”. Direct effects of NO include all those reactions where it interacts with the biological target, while the indirect ones are mediated by the RSNO mainly deriving from its interaction with O_2_ or O_2_^−^. Furthermore, direct effects require low concentrations (range of nM) of NO while indirect effects occur under high concentration levels (μM-mM range). One example where NO influences oxidative/nitrosative stress functioning as “antioxidant” could be related to enzymatic inhibition of the ROS producing enzymes NADPH oxidases probably via protein nitrosylation followed by loss of the heme prosthetic group [41]. It has been reported that high production of NO by activated macrophages inhibits the formation of O_2_^−^ generated by NADPH oxidase. The authors suggest that this event is fundamental to buffer high production of peroxynitrite thus avoiding indiscriminate oxidation reactions [42]. Oxidative stress can also induce the transcription of the gene encoding EGR-1 (early growth response-1), a process required for macrophage differentiation. It was shown that NO produced by iNOS is able to inhibit the up-regulation of this pathway in rat lung macrophages activated with lipopolysaccharides (LPS) and interferon-gamma (IFN-γ), functioning as an immunosuppressor [43].

Mild nitrosative stress can also be part of a signaling process responsible for antioxidant cell response upon GSH modulation. In particular, we demonstrated that decrement in GSH level, induced pharmacologically by treatment with buthionine sulfoximine (BSO) or metabolically by fasting, resulted in increased NO bioavailability at the muscle level, a condition that could be detrimental [44]. However, we showed that the increase of NO resulted in positive regulation of p53, which potentiated the antioxidant defense response mediated by NFE2L2 through direct upregulation of Peroxisome proliferator-activated receptor gamma coactivator 1-alpha (PGC-1a) [44,45]. On one side, this signaling axis could explain the beneficial effects mediated by exercise and caloric restriction in young mice and, on the other side, it could represent a target for determining the adaptive response of skeletal muscle under aging or pathological conditions. In fact, in the last years considerable attention on the hypothesis that increased levels of ROS characterize the development of muscle dysfunction during aging is acknowledged, due to several data showing increased oxidative damage to lipids, DNA and proteins in isolated skeletal muscle fibers from old mice [8]. Moreover, the GSH/GSSG significantly differs between young and old mouse muscles, with a significantly lower value in old animals. Although skeletal muscle is particularly equipped with antioxidants systems that are deputed to counteract ROS/RNS-mediated oxidative stress, these systems may become defective with aging or in related pathologies and interventions aimed at preserving antioxidant response are inhibitory of some muscle age-related dysfunctions [46].

## 3. GSH/NO Crosstalk in Skeletal Muscle Physiology

Skeletal muscle represents the most important district of the body where fluctuations of ROS/RNS could be observed in relation to physiological function. Muscle cell homeostasis is strictly dependent on regulated NO production by nNOS localized at the acetylcholine receptor [47]. Consistently, NO is involved in myotube differentiation and synaptic signaling from nerve to myotube. An increase in ROS production also occurs during differentiation due to the dramatic increase of NADPH oxidases activity [48]. On the other side, myoblasts derived from GPx1 knockout (Gpx1-/-) mice scarcely differentiated leading to only a few myotubes with respect to wild-type cells [49].

Physical activity of the skeletal muscle is directly correlated with O_2_ consumption, therefore, more intense is the exercise greater is the production of ROS/RNS. Moreover, such changes could be very fast due to conversion from rest to intense muscle contraction. This could explain the various and efficient antioxidant systems at muscle level including various isoenzymes of Superoxide dismutase (SOD), GPx and catalase, high activity of GSH-dependent enzymes and a strong ability to synthesize new GSH [24,50]. Additionally, GSH levels in skeletal muscle are related to the metabolic profile of the tissue; in healthy human skeletal muscle fibers, the level of GSH is higher in aerobic type I fibers than in anaerobic type II fibers [51]. There are several potential sources of ROS/RNS in skeletal muscle but also other organs can participate in their production during exercise. However, because of the invasive nature of analyses used to determine antioxidants variations, the majority of the studies during exercise in humans evaluated blood markers, whereas data at muscle level were mainly from animals exposed to exercise training. As generally accepted, mitochondria represent the major source of ROS, but during exercise at skeletal muscle this is still debated since mitochondria produce more ROS under state 4 (basal) with respect to state 3 (maximum rate of ADP-stimulated respiration), the latter being the condition under aerobic muscle contraction [52]. Additional sources, as above mentioned, include NADPH-oxidase enzymes located at the different membrane structures (sarcoplasmic reticulum, plasma membrane, transverse tubule), phospholipase A2 and xanthine oxidase [50]. Changes in expression/activity of antioxidant enzymes, such as SOD and GPx, were deeply investigated both in humans and animals during training; their increases are muscle-specific with higher effect in oxidative fibers also at mitochondrial level [24,51]. Controversial results were reported for catalase activity because the majority of the studies in humans determined the level of H_2_O_2_, a very unstable substrate. However, catalase was found to be increased in the gastrocnemius of mice upon prolonged exercise [53]; instead, GSH was always affected in all muscle types and increased levels of the tripeptide characterized trained muscle [54]. Recently, a metabolome approach was used in order to characterize the effects of exercise on human skeletal muscle. The results showed that vigorous exercise leads to GSSG and RSSG production as a consequence of increased oxidative stress [55].

Behind the detrimental role of excessive ROS, recent evidence has documented that low ROS levels produced during exercise have positive effects by influencing cellular adaptation [56]. Protein S-glutathionylation represents a reversible and ubiquitous process important for avoiding excessive oxidation of thiol-derivatives of protein cysteine under oxidative stress. The glutathionylation process is mediated by Trx or other “redoxins” and is responsible for protein structure/function changes, therefore, resulting in the modulation of several cellular metabolic processes [4]. Heart and skeletal muscle contraction and metabolism are regulated by such process through modulation of several ions pumps and contractile proteins [57,58]. In fact, mammalian fast-twitch (i.e., type II) muscle fibers exposed to GSH and reactant to induce S-glutathionylation showed an increase in their sensitivity to Ca^2+^, which was abrogated by the reducing agent dithiothreitol (DTT), indicating not only modification of protein thiols, but also that glutathionylation of skeletal muscle proteins can modulate Ca^2+^ sensitivity [59]. Moreover, the authors reported that the protein responsible for such an effect was the fast troponin I, which can be S-glutathionylated on Cys134. The most intriguing evidence concerning this protein is that the same Cys residue can be also S-nitrosylated with a concomitant decrease in Ca^2+^ sensitivity. This study provides unambiguous evidence on the cross-talk between GSH and NO in muscle functionality: NO counterbalances the ability of oxidant-induced S-glutathionylation to increase Ca^2+^ sensitivity, a condition that if persistent would adversely affect skeletal mass performance [60]. Skeletal muscle mitochondria subjected to in vitro treatment with compounds inducing S-glutathionylation (diamide or disulfiram) produced less ROS as a by-product of aerobic oxidation of either carbohydrates or fatty acid derivatives. This inhibition was due to impaired pyruvate uptake by deactivation of mitochondrial pyruvate carrier and S-glutathionylation of Complex I [61]. This regulatory process is fundamental in avoiding increased ROS burst that can result in irreversible protein oxidation and mitochondrial damage. The GSH mediated reversible oxidation of specific proteins directly related to ROS production could have a twofold benefit: on one hand it lowers the harmful effects of high ROS flux and on the other hand it allows the restoration of protein functions once the intracellular GSH content is reestablished.

As before mentioned also NO has a key role in skeletal muscle as it is involved in several functions including contractility and blood flow. Straightforward evidence of NO requirement in muscle physiology was obtained from experiments with NOSμ-/- animals where alterations in mitochondrial morphology, bioenergetics and unfolded protein response were demonstrated [62]. These modifications produced skeletal muscle dysregulation of growth and exercise performance. Generally, NO inhibits force production and modulate Ca^2+^ release by direct interaction with sGC and ryanodine receptors, respectively [63,64,65]. Moreover, it inhibits the activity of Ca^2+^-dependent sarcoplasmic reticulum (SR) ATPase to avoid the depletion of SR Ca^2+^ [66]. NO avidity to react with heme, the place where also oxygen binds, makes these two gaseous molecules in competition especially into the mitochondria, where high content of heme proteins and Fe-S groups are present to reduce the molecular oxygen concomitantly with ATP production. Indeed, the most recognized effect of NO on skeletal muscle metabolism is its capacity to directly inhibit mitochondrial electron transport chain complexes: NO inhibits electron transfer and NADH-dehydrogenase function at the level of Complex I through the intra-mitochondrial production of ONOO^·−^ [67]; NO inhibits electron transfer at Complex III independently of oxygen concentration by inhibiting the transfer of electrons to cytochrome *c* and increasing the production of O_2_^−^ [68]; NO inhibits cytochrome *c* oxidase activity in dependence of oxygen concentration and at the same time NO can be a good substrate for the enzyme. Then cytochrome *c* oxidase engagement with NO can be inhibitory of cellular respiration or for removal of NO from the cell [67,69]. At cellular level NO promotes muscle homeostasis by stimulating glucose transport during exercise trough activation of upstream signaling events leading to the translocation of glucose transporter GLUT4 at the cell surface [70]. One of the demonstrated mechanisms relies on the increase of cGMP level concomitantly with the activation of sGC during exercise. Chemical inhibition of NOS or nNOS knockout in mice muscle abolished such effects while NO donors raise cGMP level and glucose uptake [71].

NO modulates mitochondrial biogenesis through different routes, a selective one is by increasing the phosphorylated-active form of AMP-activated protein kinase (AMPK) that in turn induces PGC-1a signaling pathway [72]. PGC-1a is a nuclear and mitochondrial transcriptional coactivator inducing the expression of a large set of genes in response to metabolic and physiological stimuli, such as physical exercise [73,74]. Another pathway is shared with ROS by the activation of NFE2L2 expression, which is the master regulator of antioxidant response upon oxidative burst and mitochondrial biogenesis upon exercise in muscle [75]. NO modulates mitochondrial biogenesis also during myocytes differentiation by S-nitrosylation-mediated activation of the transcription factor CREB (cAMP response element-binding protein), which efficiently binds to the PGC-1a gene promoter region increasing its concentration and downstream signaling events [76]. Finally, NO produced by the endothelium affects skeletal muscle functions besides its canonical role in the regulation of vascular tone [77]. In fact, eNOS knockout mice (eNOS-/-) showed a reduction of mitochondrial complexes I and ATP synthase and altered skill in physical exercise: glucose and long-chain fatty acid uptake and glycogenolysis were markedly accelerated [78].

**Figure 3:**
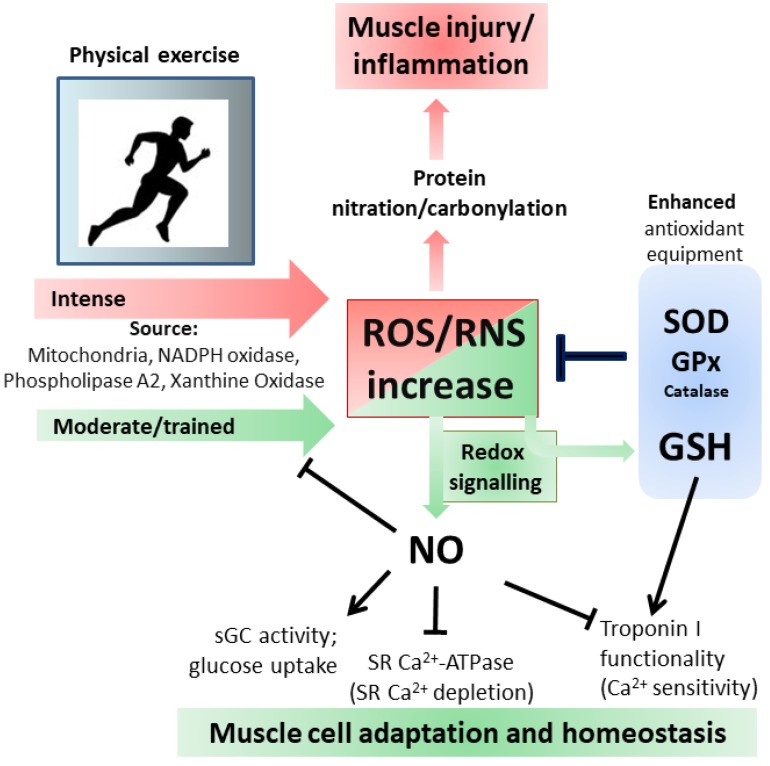
Oxidative and Nitrosative stress/signaling during intense and moderate physical exercise. For details see the text.

Through the formation of ONOO^·−^ or ONOO^·−^-derived radical (NO_2_), NO is indirectly responsible for irreversible damage to protein residues such as tyrosine nitration. This modification can result in dramatic changes in the target protein structure/function with loss of the activity or acquisition of additional abnormal role. Moreover, phosphorylative cascades can be altered being tyrosine a residue directly involved in phosphor-mediated signaling pathways and the generation of antibodies against nitrated proteins induces immunological responses [79]. Protein carbonylation, which occurs on arginine, lysine, proline and threonine, represents the most widely studied marker for oxidative damage to proteins by high ROS levels; both carbonylated and nitrated proteins are accumulated in different types of muscle during aging or related pathologies [80,81,82] (Figure 3).

Considering the importance of GSH and NO in the control of muscle responsiveness, nutritional approaches aimed at increasing their concentrations have been tested to preserve muscle homeostasis. For instance, long-term supplementation with a cystine-based antioxidant compound is able to delay age-associated muscle loss [83]. Direct supplementation of GSH precursors, including N-acetylcysteine, L-alanine and L-glutamine, was also shown to promote muscle performance and recovery during exercise [84,85,86]. Nevertheless, the intake of antioxidants such as Vitamin C and E impedes a number of physiological responses activated during muscle training with no effect on GSH production [87,88]. This suggests that the promotion of GSH metabolism rather than blunting ROS production alone is the way for assuring adaptations to exercise [89]. On the other side, nutritional supplementation of the NO precursors L-Arginine and L-Citrulline has been clearly shown to ameliorate muscle homeostasis in terms of improved protein synthesis rates, myotube diameter and muscle antioxidant capacity [90,91]. These phenotypical advantages were also ascribed to the induction of iNOS expression and appreciable in wasting conditions obtained by deprivation of growth factors and/or nutrients.

### 3.1. GSH and NO in Skeletal Muscle Inflammation During Aging

Muscle inflammation refers to a condition where local injury (e.g., disruption of blood vessel integrity or tissue damage) releases molecules that activate sentinel cells able to mount an inflammatory response. These early mediators of inflammation released by resident cells facilitate the recruitment of inflammatory cells from blood, mainly neutrophils followed by monocytes that are converted into macrophages at the muscle level. Neutrophils are deputed to clean the injured zone through phagocytosis and release of proteolytic enzymes and ROS/RNS. During this process neutrophils, macrophages and resident cell types secrete cytokines for cell signaling. Furthermore, infiltrating macrophages, cytokine production and inflammatory pathways are systemically increased during aging (inflammaging) also at the skeletal muscle level with a concomitant alteration of redox-mediated signaling pathways and muscle loss [92,93,94].

Several data are available indicating that the overproduction of NO and iNOS is responsible for inflammatory myopathies, muscle wasting, cachexia, muscle dystrophy and injuries [95,96,97]. Consistent with these findings, NOS inhibitors have been shown to reduce skeletal muscle inflammation and necrosis [98]. Among the several molecular factors involved in inflammatory response the NAD^+^-dependent histone deacetylase Sirtuin 1 (SIRT1), p53 and nuclear factor kappa-light-chain-enhancer of activated B cells (NF-κB) play pivotal roles in conditions characterized by nitrosative stress. It has been reported that the skeletal muscle of aged rats shows increased levels of iNOS, S-nitrosylated SIRT1 and acetylated p53. The treatment of aged rats with an inhibitor of iNOS or the use of GSNO in vitro demonstrated that SIRT1 is inhibited by S-nitrosylation and this results in p53 and NF-κB increased activity with consequent production of pro-inflammatory cytokines [99]. In this regard, our in vitro experiments evidenced that under GSH depletion the increased NO availability induced S-nitrosylation of nuclear resident p53 at different cysteine residues, among which the Cys124 was essential for the induction of p53-mediated antioxidant response in C2C12 myoblasts. This process was impaired during aging due to a decreased localization of nNOS at the nuclear level [45]. Notably, the occurrence of inflammatory conditions characterized by increased NF-κB activation and the consequent production of TNF-α, IL-1β and IL-6 in murine C2C12 myotubes was abrogated by treatment with a molecule able to boost intracellular GSH content [100]. These results suggest that compounds able to buffer GSH decrement could be promising candidate drugs to prevent or treat the onset and progression of inflammatory condition/diseases (i.e., aging, atrophy, cachexia, sarcopenia). Additionally, our in vivo experiments confirmed low levels of GSH in skeletal muscle of aged mice with a concomitant accumulation of carbonylated/nitrosylated proteins, as an index of oxidative/nitrosative stress, and an increased level of muscular atrophy markers and of pro-inflammatory cytokines. We also reported that aged skeletal muscles display a progressive decline of adipose triglyceride lipase (ATGL), which could be responsible for increased intramyocellular lipid droplets [101]. ATGL is expressed in type I (oxidative) muscle fibers and catalyzes the first step of triacylglycerol degradation; released free fatty acids are used both for oxidative mitochondrial energy production and as important signaling molecules for the family of peroxisome proliferator-activated receptors (PPARs) [102,103]. Among the molecular factors belonging to this family PPARα is considered a powerful repressor of inflammatory genes [103,104]. The role of ATGL in orchestrating the anti-inflammatory response was demonstrated by experiments carried out in young ATGL-/- mice, which recapitulate the same changes observed in skeletal muscle of wild-type old mice. These results clearly indicated that free fatty acids generated by ATGL lipolytic activity are fundamental for signaling the antioxidant response mediated by NFE2L2 in skeletal muscle through PPARα [101]. Moreover, the signaling axis alterations observed during aging are associated with muscle dysfunction and therefore could represent potential targets for therapeutic intervention. Similar results were reported in experiments of skeletal muscle regeneration after injury where NEF2L2 deletion results in alteration of antioxidants and delayed muscle cells response. Indeed, NFE2L2 gene disruption determined an increase in ROS, ubiquitinated proteins, inflammation and a decline in GSH [105]. Another study also proved a significant increase in ROS production and protein carbonylation together with a decrement of antioxidants in skeletal muscle of NFE2L2-/- mice. However, the exacerbation of age-related mitochondrial oxidative stress did not affect the decline of skeletal muscle respiratory function confirming that NFE2L2 is not exclusive but part of signaling pathways that are deranged during aging [106].

An aspect that is acquiring relevance in muscle physiology is the “endocrine” nature of this organ by releasing an array of muscle-derived signaling molecules (myokines) that include irisin and myostatin, cytokines and receptor antagonists known to communicate with other organs and to regulate myocytes proliferation and differentiation [107,108]. Although this issue is far from the main focus of this review, evidence on the role of oxidative/nitrosative stress in this context is emerging. Indeed, IL-6 was highly induced when GSH levels were significantly decreased in the soleus of rats exposed to a short-term high-fat diet [109]. In line with this, a ROS/RNS-dependent regulation of IL-6 was envisaged in response to muscle exercise as shown by an impaired release of this cytokine after administration of vitamins A, C and E or inactivation of NOS by N(ω)-nitro-l-arginine methyl ester (L-NAME) [110,111].

### 3.2. Inflammation and Exercise

Inflammation at the muscular level is also observed after physical exercise and if it is performed vigorously can be used as a valuable model for trauma-induced muscle damage. Strenuous exercise provokes severe muscle damage characterized by ultrastructure changes, macrophages infiltration, inflammatory cytokines production and oxidative stress paralleled by GSH decrement and increased GSSG production [55,112]. This sequence of events was demonstrated in a double-blind, crossover design study performed with healthy man volunteers [86]. In this study, the role of GSH supplementation by the antioxidant N-acetylcysteine was also investigated. However, the results obtained demonstrated that even though the antioxidant inhibited GSH decrement and attenuated the inflammatory response, the skeletal muscle suffered for efficient recovery from the damage, suggesting that blunting the redox-mediated signaling pathways is not beneficial. Among the several processes involved in muscle damage and recovery, the regeneration phase could be impaired at different levels by antioxidants, e.g., satellite cell proliferation is positively modulated by ROS produced by increased NADPH oxidases via the protein kinase B(Akt)/MyoD pathway [113]. The redox-dependent regulation of this pathway was also demonstrated in animals [114]. These results suggest that a moderate but not prolonged exercise should be encouraged in order to lower inflammatory levels and pointed out the great importance in the identification and characterization of the mechanisms that underlie the beneficial effect of exercise on health as extraordinary tools for personalized medicine [115,116,117].

### 3.3. Inflammation and Atrophy

Increased oxidative/nitrosative stress and inflammation are also features of inactivity-mediated skeletal muscle atrophy [118,119]. Atrophy is defined as a reduction in muscle mass due to disease or disuse characterized by an altered protein turnover with increased degradation with respect to synthesis. The physical inactivity induces protein ubiquitination and autophagy biomarkers [120]. Atrophic muscles were characterized by a significant increase in TNF-α, NF-κB, IL-6 levels [121,122] and in ROS production by dysfunctional mitochondria [123]. The cytokines produced during long periods of inactivity determine, in turn, NF-κB-mediated transcription of iNOS and the production of ROS/RNS at the mitochondrial and cytosolic level [124,125]. Increased NO flux can cause modification on tyrosine residues that induced the ubiquitin-proteasome pathway enhancing skeletal muscle apoptosis and atrophy [126,127]. Experimental bed rest for 35 days induced oxidative damage and cytokine production in human *vastus lateralis* fibers and significant muscle mass reduction. In addition, a significant increase in GSH availability was determined and considered as an antioxidant response to a previously elicited ROS/RNS production [128]. Increased activity in xanthine oxidase was reported as a cause of the increased level of ROS in soleus muscle atrophy induced by hindlimb unloading for 14 days. The molecular mechanism depicted was via activation of Mitogen-activated protein kinase p38 (p38 MAPK) and the E3 ubiquitin ligase Atrogin1/MAFbx, the latter being up-regulated during immobilization also in humans and involved in increased proteasome activity [129].

**Figure 4:**
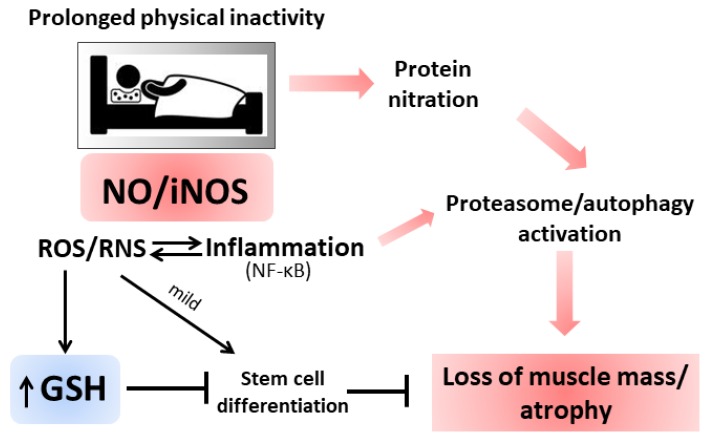
Oxidative/Nitrosative stress during prolonged physical inactivity. For details see the text.

The sources of radical species under atrophy are still not completely identified as well as the chronology of the events between oxidative stress and inflammation. Several hypotheses were depicted and some still contradictory: Suzuki et al., reported that prolonged inactivity in limb skeletal muscles (i.e., 14 days of hindlimb suspension) is accompanied with increased NO levels in the inactive muscle without a concomitant increase in iNOS activity [130]; another study reported that 14 days of hindlimb suspension determined a decrease in both muscle nNOS and NO levels [131]. However, increased NO levels could mediate the activation of Ataxia telangiectasia mutated kinase (ATM) and Liver kinase 1 (LKB1) pathways, which by stimulating AMPK resulted in mTORC1 repression and protein synthesis decline [132]. Nitrosative stress can also accelerate the atrophy process by stimulating inflammatory gene expression and increasing the abundance of key proteins involved in proteolytic systems. Thus, NO could modulate muscle atrophy via redox-mediated control of protein synthesis, proteolysis or inflammation and GSH is of fundamental importance to counterbalance high nitrosative stress that whether exacerbated could result in cell death. On the other hand, the high GSH concentration observed under atrophy could impede the redox-mediated signaling pathways necessary for muscle homeostasis (i.e., aged differentiated muscle cell replacement by stem cells that assures muscle functionality). Nonetheless, from the several available data, it is possible to assert that the relationship between ROS and atrophy is well-grounded and future research clarifying the connections at the molecular level will be fundamental for the identification of molecular targets for therapeutic interventions (Figure 4). In addition to the role played by NO in muscular atrophy, experiments with NOS inhibitors have also demonstrated a reduction of muscle hypertrophy in association with an increase in slow myosin heavy chains and α-actin expression [133,134,135]. The molecular mechanism mediated by NO consists of the activation of G0-satellite cell proliferation: Released NO crosses the sarcolemma and stimulates hepatocyte growth factor (HGF) activity. HGF interacting with c-met receptor of satellite cells promotes their entrance into the cell cycle [135,136]. Through this mechanism, NO works as an activator of hypertrophy in endurance or stretching sports.

## 4. Conclusions

Tight-regulated crosstalk between NO and GSH is pivotal for maintaining muscle homeostasis, as they regulate a variety of signaling mechanisms necessary for preserving muscle integrity under resting and physical exercise stimulation. During the last years the discovery of radical sources, the understanding of the mechanisms and the consequences of their production on muscle homeostasis is expanded exponentially also thanks to new research tools and techniques. Starting from the damaging effects of radical species that formerly gave the ascertain of their involvement in muscle functions we now have the knowledge that ROS/RNS are fundamental for proper muscle physiology, as part of redox-mediated signaling pathways, and that derangement in their production participates in muscle aging and dysfunctions. These aspects were the main topics of this review. Being the most abundant tissue in the human body, skeletal muscle represents the major site for metabolic activity; muscle serves as a reservoir of proteins and therefore a source of amino acids and as a metabolic regulator for glucose and lipid homeostasis. In this regard, exercise could enforce improvements of functions in other organs by ameliorating muscle metabolism and counteracting atrophy. However, the molecular mechanisms underlying muscle contraction and organ fitness are still largely unknown even though very recent discoveries pointed out for the role of mitochondria activities and morphology in starting signaling processes. A key issue that needs careful validation is the use of direct antioxidants (e.g., vitamins) during exercise training as they seem to blunt the redox-mediated signaling necessary for muscle adaptations. On the contrary, dietary supplementation of GSH or NO precursors seems to benefit muscle recovery and homeostasis. This supports the importance of GSH/NO pathways in muscle physiology and suggests that their promotion by nutritional strategies is a way to ameliorate physical wellness. Future investigations need to provide details about the crosstalk between NO/GSH and the endocrine function of muscle since the production and release of myokines place this organ central to the physiology of metabolic regulation.
